# AAPM task group report 305: Guidance for standardization of vendor‐neutral reject analysis in radiography

**DOI:** 10.1002/acm2.13938

**Published:** 2023-03-30

**Authors:** Kevin Little, Ingrid Reiser, Bruce Apgar, Poonam Dalal, Jaydev Dave, Ryan Fisher, Katie Hulme, Mary Ellen Jafari, Emily Marshall, Stephen Meyer, Quentin Moore, Nicole Murphy, Thomas Nishino, Katelyn Nye, Kevin O'Donnell, John Sabol, Adrian Sanchez, William Sensakovic, Lawrence Tarbox, Robert Uzenoff, Alisa Walz‐Flannigan, Charles Willis, Jie Zhang

**Affiliations:** ^1^ Ohio State University Columbus Ohio USA; ^2^ The University of Chicago Chicago Illinois USA; ^3^ AGFA HealthCare Greenville South Carolina USA; ^4^ GE Healthcare Chicago Illinois USA; ^5^ Thomas Jefferson University Philadelphia Pennsylvania USA; ^6^ The MetroHealth System Cleveland Ohio USA; ^7^ Cleveland Clinic Cleveland Ohio USA; ^8^ Atlantic Health System Morristown New Jersey USA; ^9^ Canon Medical Components USA Irvine California USA; ^10^ Mercy College of Ohio Toledo Ohio USA; ^11^ Ann & Robert H. Lurie Children's Hospital of Chicago Chicago Illinois USA; ^12^ University of Texas MD Anderson Cancer Center Houston Texas USA; ^13^ Canon Medical Research USA Vernon Hills Illinois USA; ^14^ Vanderbilt University Nashville Tennessee USA; ^15^ Mayo Clinic Phoenix Arizona USA; ^16^ University of Arkansas for Medical Sciences Little Rock Arkansas USA; ^17^ Fujifilm Medical Systems USA Lexington Massachusetts USA; ^18^ Marshfield Clinic Marshfield Wisconsin USA; ^19^ University of Kentucky Lexington Kentucky USA

**Keywords:** Computed radiography, digital radiography, radiography, reject, reject rate analysis, reject rate, repeat

## Abstract

Reject rate analysis is considered an integral part of a diagnostic radiography quality control (QC) program. A rejected image is a patient radiograph that was not presented to a radiologist for diagnosis and that contributes unnecessary radiation dose to the patient. Reject rates that are either too high or too low may suggest systemic department shortcomings in QC mechanisms. Due to the lack of standardization, reject data often cannot be easily compared between radiography systems from different vendors. The purpose of this report is to provide guidance to help standardize data elements that are required for comprehensive reject analysis and to propose data reporting and workflows to enable an effective and comprehensive reject rate monitoring program. Essential data elements, a proposed schema for classifying reject reasons, and workflow implementation options are recommended in this task group report.

## 1 INTRODUCTION

### 1.1 Charge

This report constitutes a consensus‐based guidance document that recommends standard data elements and an effective dataflow to enable a vendor‐neutral reject analysis program in radiography. These recommendations expand upon the suggestions of the American Association of Physicists in Medicine (AAPM) Report 151 to include frameworks for implementing a robust reject analysis program through data standardization.

### 1.2 Scope

This report concerns general radiography, including stationary, mobile, portable, and digital retrofit units. It does not apply to screen‐film radiography or any type of mammography.

### 1.3 Background

In diagnostic radiography, radiation exposures of patient anatomy that result in patient dose but do not produce an image that is presented to the radiologist for diagnosis are called *rejects*. ***Rejected radiographs*** cause unnecessary patient radiation dose. In screen‐film radiography, the definition of a rejected radiograph also included wasted films and non‐patient imaging such as QC images,^1^ but this report only considers patient images that were not used for diagnosis, consistent with the American Association of Physicists in Medicine (AAPM) Report 151.

**FIGURE 1:**
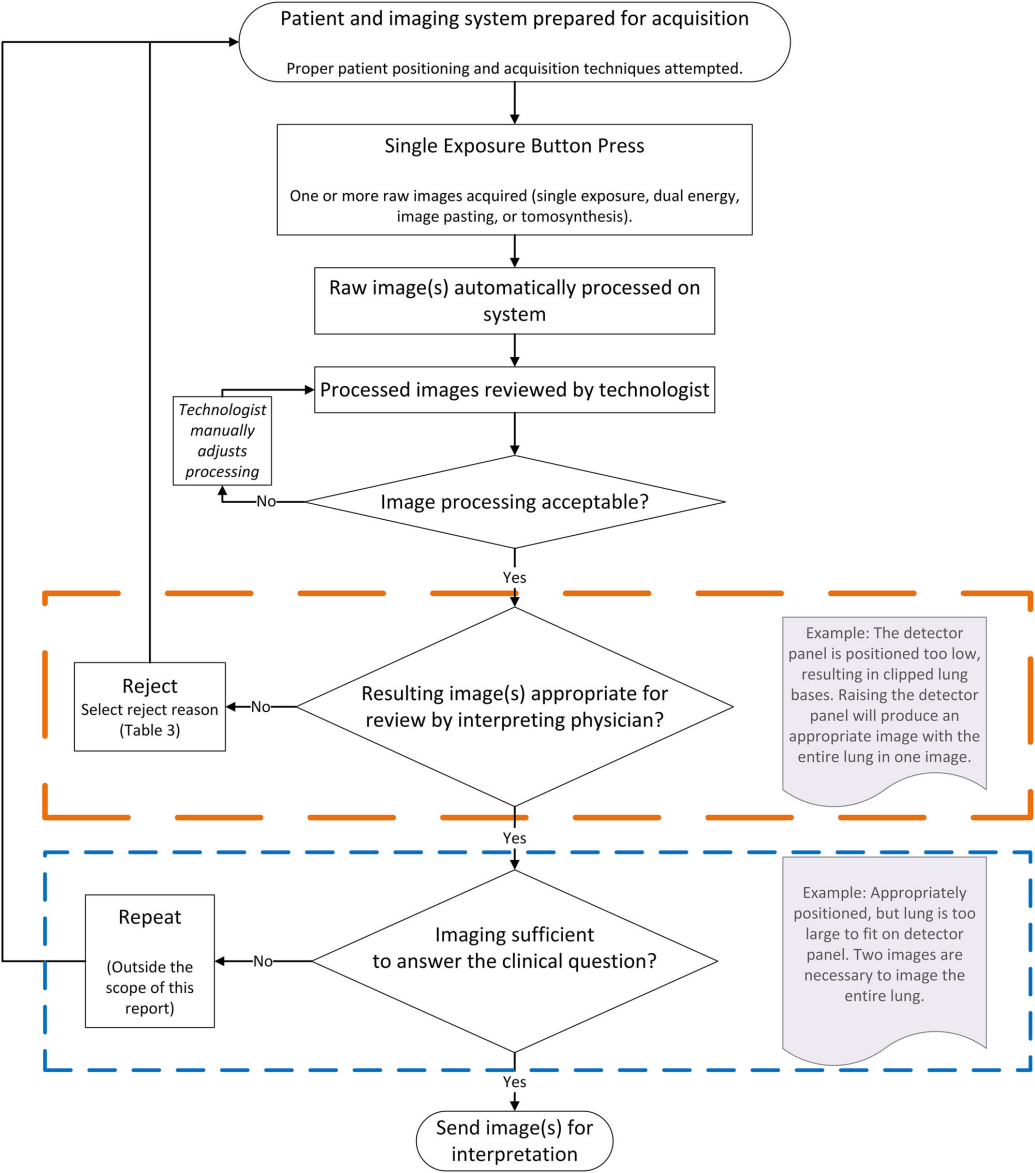
Generic actions and decisions taken by the radiologic technologist when performing radiographic imaging.

***Repeat radiographs*** exceed the number of expected images for a given exam or view. As opposed to rejects, repeat radiographs are sent to long‐term storage, such as a picture archiving and communication system (PACS), and are reviewed by a radiologist. There might be valid reasons for radiologic technologists to acquire repeat radiographs, such as when body habitus unexpectedly exceeds the detector size—for example, when the patient's lung field is larger than the detector panel, requiring two radiographs to depict the entire lung anatomy. However, there should be clear protocols in place to limit the number of repeats to those specific situations in which a standard anatomic view is insufficient. Radiographers must follow these protocols and standards set by their departments. Radiographers should accurately identify and document reasons why images are repeated.^2^ Repeat radiographs may also be explicitly requested by the radiologist to meet specific clinical requirements. Repeat radiographs are outside the scope of this report. Figure 1 outlines the steps that a technologist takes to acquire images, including decisions that may produce rejects and repeats.

Reject rate analysis is considered an integral part of a radiographic quality control (QC) program.^1^, ^2^, ^3^, ^4^, ^5^ While the dose in radiography is generally low, according to the AAPM, “imaging procedures with ionizing radiation should be used when clinically appropriate and be performed using the lowest radiation dose necessary to accomplish the clinical task.^6^” Reject rates that are either too high or too low may suggest systemic department shortcomings in QC mechanisms. There is considerable spread in the reject rate targets proposed by various societies. The Conference of Radiation Control Program Directors (CRCPD) suggests a range of 5%–7%,^7^ while AAPM Report 151 suggests a target of 8%, with 10% as the upper threshold and 5% as the lower threshold for investigation.^5^ This target value proposed by AAPM Report 151 is perhaps more realistic given published reject rate analyses.^8^, ^9^, ^10^, ^11^, ^12^ For pediatric clinics, AAPM Report 151 suggests a target of 5%, with 7% as the upper threshold for investigation.^5^

In the era of screen‐film, typical reject rates were found to be around 11%, with Gray reporting several studies that achieved single‐digit reject rates post‐intervention.^1^ The primary reason for rejects in screen‐film radiography was incorrect technique, with 40–45% of rejects due to over‐ or underexposure.^1^, ^13^ The substantially improved dynamic range of computed radiography (CR) detectors reduced the occurrence of over‐ and under exposure, and early experiences with CR suggested that reject analysis may not be needed in digital radiography.^14^, ^15^ Peer et al. reported a decrease in reject rate from 27.6% to 2.3% when transitioning to CR from screen‐film radiography.^14^ While reject rates were generally low with CR,^16^, ^17^ several sites have reported higher reject rates when transitioning from CR cassettes to direct digital radiography (DR) using flat panel detectors.^8^, ^9^, ^10^, ^11^, ^12^, ^18^ With the exception of Midgley, these studies report reject rates for DR in the range of 11% to 13%, emphasizing the importance of reject rate monitoring in a digital environment. In all these studies, patient positioning was found to be the primary reason for rejecting a radiograph.^8^, ^9^, ^10^, ^11^, ^12^, ^18^ Waaler hypothesizes that non‐technical reasons might have contributed to an increase of rejects in DR, such as the low perceived barrier towards “taking one more image just to be sure,” which carries a minimal workload penalty in a digital environment compared to screen‐film,^11^ or CR. Furthermore, radiologists reviewing images side‐by‐side with technologists has become rare,^2^ and as a result, radiographers might be uncertain as to the expected image quality criteria. As a remedy, there is an increased need for guidelines, in‐service education, and training.^2^, ^11^ Such activities can be more effective with knowledge of the rejected anatomies and/or views.^12^, ^19^ Analysis of a department's overall reject rates can help with process improvement and help improve the overall performance of a radiology department while minimizing patient radiation dose.^2^

In addition to monitoring reject rates, it is important to have the ability to review the rejected images, as a recent study by Rosenkrantz et al. found that radiographs are often rejected inappropriately. This study found that 93.1% of rejected chest radiographs provided sufficient diagnostic information, implying only about 7% of these radiographs needed to be rejected. For musculoskeletal radiographs, 77.9% of rejected radiographs provided adequate diagnostic information.^20^ The study authors suggest to review the questionable radiograph in the context of all radiographs acquired for the study and to have a radiologist decide whether a radiograph should be rejected.^20^ While direct radiologist involvement for each questionable radiograph may not be feasible for many practices, these findings suggest that radiologists need to work closely with technologists to clearly define rejection criteria.^21^

As already described in AAPM Report 151, reject analysis is an important aspect of radiographic QC.^5^ Experience in implementation of AAPM Report 151 recommendations suggest that additional required information, data standardization, and a dataflow implementation framework would be highly beneficial toward making reject analysis a more efficient and useful activity.

### 1.4 Motivation

Most, but not all, manufacturers provide some form of reject analysis on their radiography systems, although often as a non‐standard option that must be additionally purchased. These analyses generally produce reports that include total reject rates. Depending on the manufacturer, these reports may offer additional information such as a breakdown of reject reasons or number of rejects per technologist, exam, or view. Due to the lack of standardization between vendors, these summary reports often cannot be easily compared between units. Even within a single vendor, most summary reports reflect reject data from a single acquisition workstation and are not configured to combine data from multiple units or locations in order to gain a global view of clinical practice.

In addition to these summary reports, radiography systems can generate log files that contain more detailed exam information, though the information provided and ease of accessibility to that information varies substantially between manufacturers. For quality review, these logs serve multiple purposes, including review of technique factors and exposure indices. Some vendors provide exposure information and reject information as separate files, without a unique link between entries, making subsequent analyses difficult, if not impossible.

**FIGURE 2:**
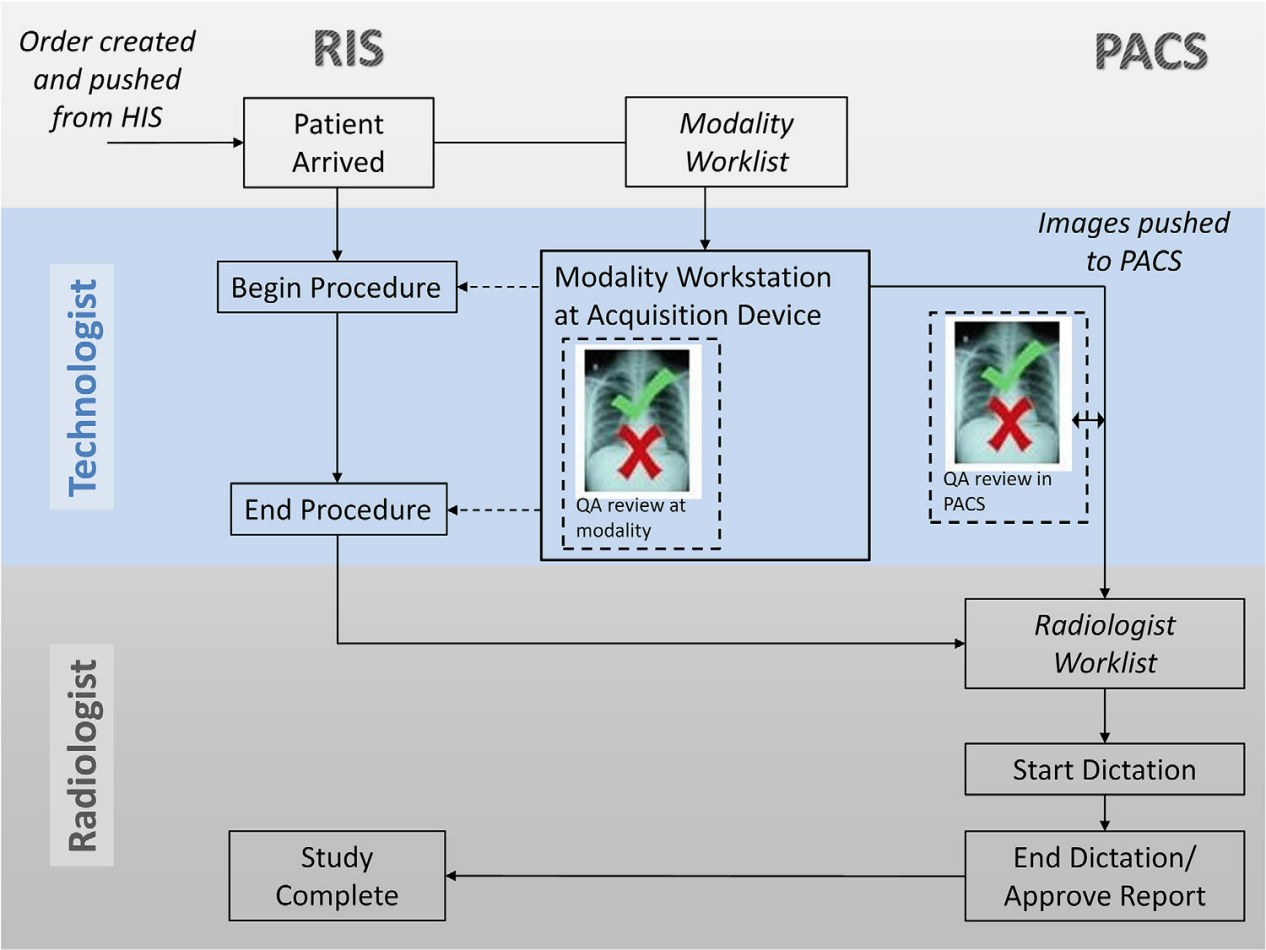
The diagram depicts a typical radiology information system (RIS)‐driven workflow for a radiology clinic. Typically, after the order has been created in the hospital information system (HIS) and pushed to the RIS, the technologist begins and ends the procedure, as indicated by the dashed arrows. Images are commonly reviewed for image quality at the acquisition workstation by the technologist that acquired the images, and images deemed “unacceptable” for interpretation are rejected and do not get sent to picture archiving and communication system (PACS). Additional technical items may be assessed in PACS during a “QA Review” stage prior to ending the procedure in the RIS. PACS super‐users have the ability to delete images from PACS during “QA Review.” Note that the radiologist has no knowledge or input to the image QA review process in this workflow.

**FIGURE 3:**
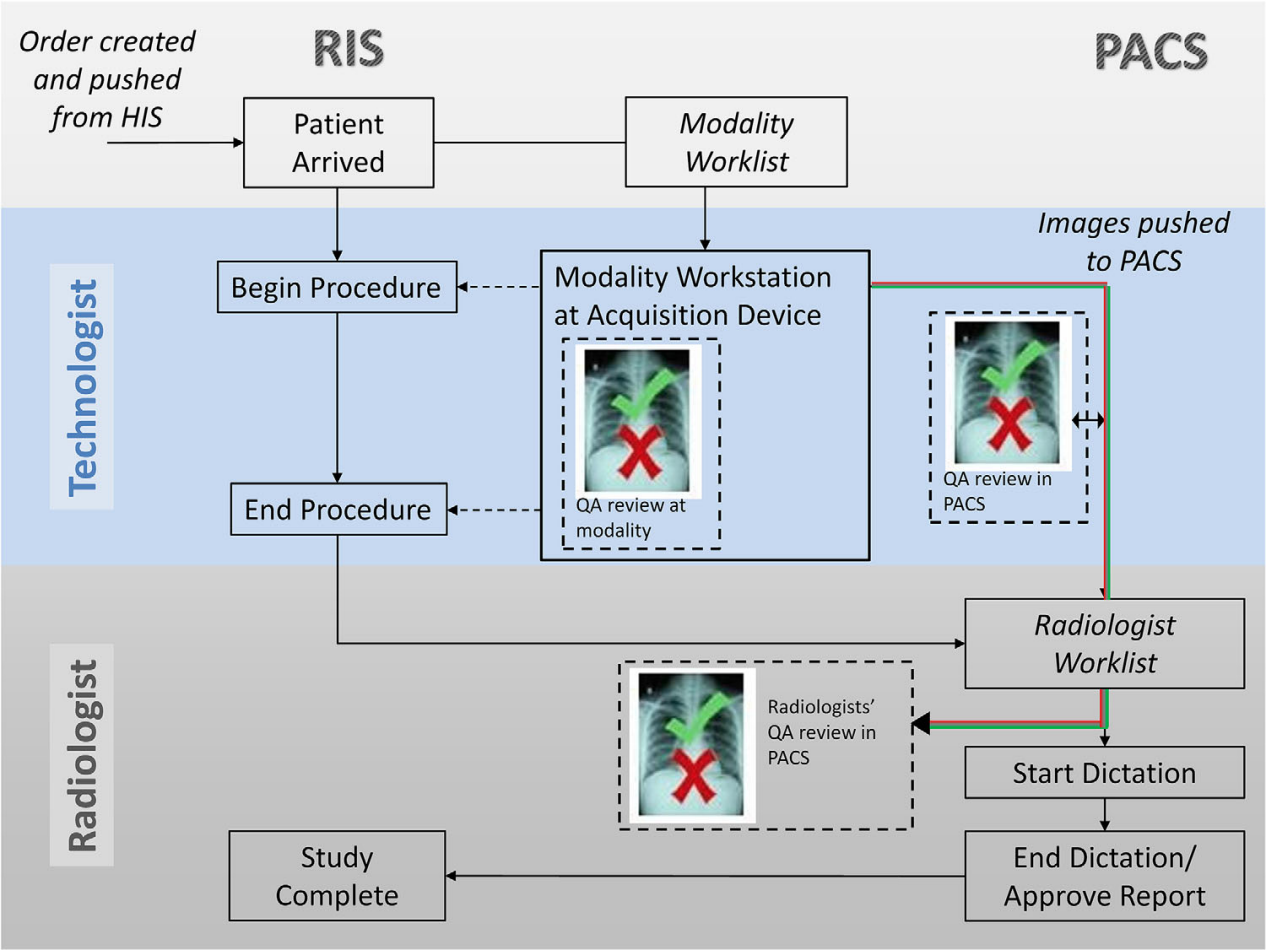
The proposed DICOM implementation would enable radiologists’ direct involvement in the image quality control (QC) review process. PACS, picture archiving and communication system; RIS, radiology information system.

Not all vendors allow for the retrieval of rejected radiographs. Rejected radiographs should always be available for review, or at least until reject analysis has been completed. While availability of the full rejected DICOM image, or a compressed thumbnail image, is beneficial, a better solution would be a framework that links the rejected radiographs with the exam. This would potentially facilitate access and review of rejected radiographs by radiologists or QC technologists at other points in the workflow (see Figures 2 and 3). The inclusion of radiologists in the process is important as they interact with technologists to provide training or other image quality‐related feedback. While currently available workflows generally include exposure information, the radiologist does not know whether multiple images were acquired and rejected. However, their judgment of the quality of the exam in terms of technologists’ skills should include the number of attempts made by the technologist to complete the exam.

In principle, a digital environment lends itself more readily to reject analysis compared to manual collection of rejected films, but the implementation can prove difficult. Some institutions have developed DICOM or server‐based in‐house solutions.^16^, ^22^ At the time of this writing, most vendors provide reject reports. However, as there is currently no standardized reporting of reject data, different vendors use inconsistent methods for analysis and data tracking. Given that most facilities utilize radiography equipment from multiple vendors, this inconsistency makes it very difficult to conduct comprehensive reject rate analysis of an entire facility or larger enterprise.^18^

The purpose of this report is to provide guidance to help standardize data elements that are required for a comprehensive reject analysis, and to propose data reporting and workflows to enable an effective and comprehensive reject rate monitoring program.

## 2 REJECT WORKFLOW IN THE RADIOGRAPHY CLINIC

A reject workflow within the radiography clinic includes both the prospective quality assurance (QA) conducted by the technologist prior to releasing images for interpretation, as well as the retrospective analysis of rejected images.

When assessing a radiograph for image quality, factors include ensuring that the relevant anatomy has been included in the image, that the appropriate centering, collimation, and body‐part orientation have been employed, that the image is free of clinically relevant artifacts, and that the image is generally suitable for interpretation. The accuracy of annotations and placement of lead markers or skin markers must also be evaluated. These factors are most commonly evaluated at the acquisition workstation by the technologist who acquires the images.

A typical clinical workflow is shown in Figure 2. While the most prevalent clinical workflow is the rejection of images at the modality by the technologist who acquired it, at some facilities alternate workflows exist where tech supervisors or IT support personnel delete images directly from PACS. It should be noted, however, that images deleted directly from PACS are not likely to be reflected in the vendor provided reject reports which are fed only from the modality itself. Furthermore, sites should put in appropriate controls to make sure physicians do not interpret images that will not be included as part of the permanent record.

## 3 ESSENTIAL ELEMENTS FOR REJECT ANALYSIS

### 3.1 Overview of data elements

Tables 1 and 2 list the minimum information that should be recorded for all acquisitions and rejects, respectively. Each entry is given a priority score for use in reject analysis. A priority score of one indicates that the element is essential to be able to compute basic reject rates for a given radiography system and technologist. A priority score of two identifies elements that are needed to perform analyses such as a breakdown of reject rates by exam types. This type of information allows for the identification of studies and conditions for which reject rates are out of range, enabling targeted interventions. A priority score of three identifies elements that are required for detailed analysis, including protocol optimization and exposure index (EI) analysis, which are quality activities closely related to reject analysis. Vendors should always provide data for all data elements, regardless of priority score.

**TABLE 1:** Data elements required for reject analysis. While not all data elements are available on every system (e.g., source‐to‐detector distance [SID] or dose area product [DAP]), elements should be included in analysis when available. Data elements in bold‐face are PHI or may allow for patient identification when combined with other information. Priority scores indicate the type of reject analysis enabled by a given data element: (1) Overall reject rate. (2) Detailed analysis. (3) In‐depth analysis including protocol optimization.

Data field	Priority	DICOM Tag	Comments
**System identifier** (e.g., station name or device serial number)	1	Station name: (0008,1010) Device serial number: (0018,1000)	Identifies the x‐ray unit that was used to acquire the radiograph.
Operator/technologist	1	(0008,1070)	May be automatically included in the DICOM key object selection (KOS).
**Acquisition date and time**	1	(0008,0022) & (0008,0032)	
**Processing date and time**	3	(0008,0023) & (0008,0033)	
**Patient MRN**	2	(0010,0020)	
**Accession number**	2	(0008,0050)	
Anatomy	2	(0008,2218); (0018,0015)	
Protocol identification	2	(0008,1032); (0040,0275)	
View	2	(0018,5101); (0054,0220), (0054,0410)	
Patient size selection	2	Manufacturer specific	Selection in the imaging protocol, rather than actual patient size.
Acquisition type	1		Identifies whether an acquisition is a single acquisition or part of a multi‐acquisition resulting from a single exposure button press, such as dual energy, image pasting, or tomosynthesis.
**Irradiation event (exposure button press) unique ID**	1	(0008,3010)	Unique identification of all acquisitions resulting from a single continuous actuation of the exposure button.
kVp	3	(0018,0060)	Can help determine if an acquisition was rejected based on technique, and/or whether technique was changed subsequently.
μAs	3	(0018,1153)	mAs (0018,1152) does not have sufficient precision.
Exposure time	3	(0018,1150)	
mA	3	(0018,1151)	
Filter	3	(0018,1160), (0018,7050), (0018,7052), (0018,7054)	These specify filter thickness, filter type, whether or not a filter exists.
Grid use	3	(0018,1166)	
Source‐to‐detector distance (SID)	3	(0018,1110)	
Receptor: Wall stand/Table/Free Detector	3		No consistent DICOM tag.
AEC status/cell location/density or speed	3	(0018,7060), (0018,7062)	
EI^23^, ^24^	3	(0018,1411)	
Target EI	3	(0018,1412)	
DI	3	(0018,1413)	
**Detector identifier (S/N)**	3	(0018,700A)	
Collimation	3	(0018,1700), (0018,1702), (0018,1704), (0018,1706), (0018,1708), (0018,1720)	To specify collimation, the value of 0018,1700 (collimator shape) determines whether 1720 or 1702–1708 are needed.
Dose area product (DAP)	3	(0008,115E)	
Image processing parameters	3	Manufacturer specific	

**TABLE 2:** Information required for rejected acquisitions, in addition to the information listed in Table 1. Data fields in bold‐face are protected health information (PHI). A compressed thumbnail image is PHI if it contains identifiable information.

Data field	Priority
Identification as a reject	1
Reject Reason—broad category	2
Reject Reason—detailed	2
**Reject date and time**	1
**Rejected image (original or compressed thumbnail)**	2

In addition to the designation of the x‐ray acquisition as rejected, elements necessary for reject analysis include the reject reason (discussed more in Section 3.4) and a copy of the rejected radiograph. The copy of the rejected radiograph could be in the form of a link or pointer to either the original quality image or a compressed thumbnail image (see Section 3.3).

Image processing parameters can provide potentially valuable information when poor image quality is caused by image processing rather than technique factors or positioning. Image processing settings are often included in the DICOM header information, but generally in non‐public (i.e., private) data elements that are only decipherable by the vendor. Availability of processing information can help reveal instances where incorrect processing parameters may be built into the protocol by the applications specialist, for example, or improperly selected by the technologist.

The required data for analysis includes fields that contain protected health information (PHI) such as medical record number (MRN) and accession number. This task group's consensus was that such information should not be omitted from reject records that are used for local internal analyses or within a context where sharing PHI is permitted. An accession number links radiographs to a unique order and allows for review of associated images, as well as retrieval of exam‐specific information that may not otherwise be available. MRN allows rejects to be associated with patients who may be receiving frequent exams, allowing insight into imaging consistency.

### 3.2 Identifying the technologist

In the era of screen‐film, Gray discouraged identifying technologists due to anecdotal reports of individuals hiding or removing rejected films for fear of punitive actions.^1^ In today's environment, technologist‐specific reject rates should be tracked to create targeted education and enable the setting of personal performance improvement goals. Alashban et. al. found a statistically significant difference in reject rate between technologists, which they hypothesized might be due to variations in training and clinical experience.^25^ Punitive actions based on reject rates is not recommended, and consideration should be given for situations that involve students or staff training. Additionally, all data should be aggregated to analyze department‐level data and trends to determine commonalities in reject reasons to foster organizational learning opportunities.

The two primary methods to identify the technologist are (1) by linking accession number with the performing technologist, which is generally available through the HIS/RIS and (2) by recording the username that was used to login to the radiography system or the selected operator at the time the exposure was taken. The latter method is not feasible if a common login is used. If technologists login with their unique login credentials, this information should be stored in the DICOM image header or system log. In order to calculate technologist‐specific reject rates, this information is required for each acquisition regardless of its reject status. Associating radiographs with the technologist who acquired the image during analysis is difficult, particularly when PHI is omitted from system log files and login information is not recorded. In principle, technologists’ lead markers could be used to extract operator information as well, although this would potentially be time‐consuming and prone to error and is therefore not recommended.

### 3.3 Rejected images

While the actual rejected radiograph is not needed to calculate reject rates, the availability of these images is a valuable tool for validating reject reasons, designing interventions, and troubleshooting. At the onset of reject rate monitoring, and periodically throughout monitoring, rejected images for a given reject reason should be reviewed, as it is not uncommon to find that the reject reason was incorrect. When reject rates are high, it is helpful to review rejected acquisitions with radiologic technologists, either as a group or individually. This review needs to be conducted by a radiologist or the image quality‐monitoring technologist to assess whether the rejection was appropriate.

The image of the rejected acquisition available for review can either be stored in the original DICOM format or as a secondary capture, in a JPEG or similar image format. If images are stored as DICOM, we recommend two potential implementations to ensure a rejected image is not included in the clinical image workflow. When an image is rejected at the acquisition workstation, the modality assigns the image a “Key Object Selection (KOS) Rejection Note,” which is a message that compatible PACS can read to identify the image as rejected and sequester it from clinical worklists. If the PACS does not have the capability to read KOS notes, an alternate location will need to be configured for rejected images to keep them from the clinical workflow. In either case, the image should be available for downstream review and should be linked to the original exam, permitting comparison of rejected acquisitions with those submitted to the radiologist for diagnostic purposes. This can be accomplished through the KOS Rejection Note that points to the rejected image (either within the exam or at an alternate location).

### 3.4 Reject reasons

When technologists reject an acquisition, a reason for the rejection is required. These reasons can later be tallied and can help guide education and intervention efforts. The user selectable reject reasons must be specific enough to be actionable, but terminology should be easily understood by technologists for consistency and to reduce misassignment.

The list of reject reasons should be small enough to allow for a quick selection that will not impact clinical workflow, but large enough to allow for its use in tailoring specific interventions. Overlap between categories should be minimal, promoting a clear choice. For example, the current DICOM “*Rejected for Quality Reasons*” includes two reason codes, “image artifacts” and “detector artifacts,”^26^ though the differences between the two are not always straightforward and the correct identification might require system analysis by a field service engineer or a medical physicist. This task group has recommended standard terminology for reject classification that is descriptive rather than diagnostic as found in Table 3.

Some vendors provide the option to classify rejected images as “re‐processed” or “non‐clinical.” Images designated as such are not included in the reject rate calculation. The intended use of this category is for images that might be acquired with inappropriate image processing, such as by selecting the wrong anatomy, producing an image with inappropriate image processing. The technologist might change image processing parameters to produce an acceptable image, and use this category when discarding the image with the incorrect processing. However, on some radiography units, the interface allows selection of this category for any image, not just re‐processed images. This may lead to misuse.

Further, in the task groups’ experience, this option can easily lead to inconsistencies: For example, in the technologists’ view, dual energy chest x‐ray produces three images (standard chest x‐ray, soft‐tissue, and bone images), which might be generated from two x‐ray exposures. The technologist may classify these under a standard reject category (Table 3), or as a re‐processed image. Depending on the choice, this reject may not be tallied in the total count. Due to these limitations, the task group recommends against providing such an option.

These flaws can be avoided by tracking unique exposure button presses and verifying that at least one “diagnostic” image per exposure button actuation is available for review by the radiologist. This methodology is discussed in greater detail in Section 4. While this solution might be feasible for DR units, it is acknowledged that this information may not be available for CR or digital retrofit systems.

### 3.5 Proposed schema for classifying reject reasons

A schema of two levels of reject reasons is proposed in Table 3. The first level is a standardized broad categorization of reject reasons. A second, more granular level may be enabled to allow for more detailed reject reasons that may be customized to the needs of an institution.

**TABLE 3:** Proposed reject reasons at the coarse and detailed levels

Broad category	Detailed category (suggested defaults, should be customizable)	Examples (not exhaustive)
1. Patient positioning	1.1 Rotation/tilt 1.2 Anatomy cut‐off 1.3 Patient orientation	Incorrect anatomy rotation; incorrect tube angle; internal vs. external rotation. Required anatomy not visualized, anatomy obscured by collimation; detector‐tube alignment; fixation device not visible. Upright vs. supine; left‐lateral vs. right‐lateral; weightbearing vs. non‐weight bearing.
2. Patient motion	2.1 Voluntary 2.2 Involuntary	Patient moved during exposure; did not follow breathing instructions. Patient has a condition that prevents cessation of moving or compliance with respiratory directions. Pediatric patients unable to follow instructions may also be included.
3. Artifacts	3.1 Known object 3.2 Grid lines or similar artifact 3.3 Nonuniformity or defect visible	Known objects such as patient buttons, jewelry, etc.; O2 Line; positioning device, improper shield placement. Electromagnetic interference artifacts; detector artifacts such as dead pixels or lines; tube artifacts such as inverse‐pinhole.
4. Image contrast or noise	4.1 Inappropriate image contrast 4.2 Unacceptable Noise/Underexposure 4.3 Saturation/overexposure 4.4 Grid use error	Incorrect kVp; nonoptimized image processing; x‐ray technique factors. X‐ray technique factors; nonoptimized image processing Pixel clipping. Erroneous use or nonuse of grid, wrong SID.
5. Incorrect selection (protocol, detector)	5.1 Incorrect protocol selected 5.2 Detector not correctly selected or initialized	Protocol selection error (anatomy, view) Incorrect detector selected; no detector selected; bucky not pushed in far enough to initialize detector. *Note: For some retro‐fit systems, an exposure button press may not produce an image. See sec. IV.B*.
6. Wrong body part or patient	6.1 Body part imaged does not match order 6.2 Patient imaged does not match order	*When the body part or patient does not match the ordered exam, this represents a potential medical error. Ideally, these would still be sent for review and would not be rejected*.
7. Equipment issue	7.1 Equipment failed during exposure	Power failure; unexpected detector disconnection; other unexpected mechanical or software failure.
8. Practitioner‐directed		Repeated imaging while positioning devices such as feeding tubes; rejected images due to physician positioning of patient.
9. No patient exposure/test		QC images; warm‐up exposures; images obtained without exposing a patient*; non‐patient research (mummies, cadavers, etc).

The categories are meant to be descriptive of the issue without necessarily diagnosing the underlying cause. For example, “Image Contrast or Noise” is used instead of “x‐ray technique factors” or “under‐ or over‐exposure.” While technique factors may be a likely cause of poor image contrast or noise, these issues may also be caused by poor image processing. Part of detailed reject analysis should include looking for trends to identify underlying problems and potential protocol improvements, including improvements in AEC use and image processing settings. Some training may be required to ensure technologists know how to correctly categorize rejects.

Mobile radiography is used in a variety of settings, including emergency departments, operating rooms, intensive care units, and post‐anesthesia care units, to assist in the positioning of devices such as feeding tubes, intubation tubes, fixation devices, core packs, etc. In these instances, if the device is not positioned correctly per the acquired image, the physician will make adjustments to the device and then request another image be acquired—only the final image (demonstrating the final correct placement) is sent to PACS and the remainder are rejected. While these rejects could potentially be grouped under “Positioning,” they are distinctly different from that category in that the repeat is not at all due to the technologist's imaging approach—rather, the images are being used to guide a procedure and are repeated as necessary. After the procedure is completed, the extra images have no remaining clinical relevance and are rejected. A more appropriate category for these rejects would be the “practitioner‐directed reject.” Currently, this is not a standard reject category on most systems but can be created if a system allows customization.

DICOM currently offers an extensible list of reasons that images may be rejected for quality issues. A comparison of our proposed reject reasons and the current DICOM reject reasons is given in Appendix A.2.

## 4 REJECT RATE CALCULATION

The reject rate (RR) should be calculated per exposure button press, with RR = N_rej_/N_tot_ where N_rej_ is the number of exposure button presses that do not result in a diagnostic image, and N_tot_ is the total number of exposure button presses. For single‐exposure acquisitions, the number of exposure button presses is numerically equal to the number of images taken. However, for multi‐acquisitions, the system may acquire multiple x‐ray exposures during a single exposure button press. Depending on the exam type, this may be two x‐ray exposures per press (dual energy), three to five x‐ray exposures (image pasting), or dozens of x‐ray exposures (tomosynthesis). Practitioner‐directed and non‐patient exposures (categories 8 and 9 in Table 3) should not be included in the reject rate calculation.

### 4.1 Clinical scenarios

Consider the following clinical scenarios:

**Image pasting**: A clinical area performs 500 exposure button presses, of which 450 (90%) are single‐exposure and 50 (10%) are image‐pasting with four images per exposure button press. Technologists rejected 67 of the single‐exposure events and 4 of the image pasting events. The reject rate for single exposure exams is (67/450) = 14.9% and that for image pasting exams is (4/50) = 8%. The combined reject rate for these examinations is (67+4)/500 = 14.2%. However, if the reject rate is incorrectly calculated based on individual images, the lower reject rate of image pasting is weighted more strongly and the apparent reject rate is (67 + 4*4)/(450+4*50) = 12.8%.

**Dual energy**: A clinical area performs 720 exposure button presses, of which an equal amount are dual energy posterior‐anterior (PA) chest imaging and single energy lateral (LAT) chest imaging. Technologists reject 27/360 = 7.5% of PA exposure events and 54/360 = 15% of the LAT events. The average reject rate for these chest exams is (27 + 54)/720 = 11.25%. However, if the reject rate was incorrectly calculated based on individual raw images, the apparent reject rate would be (27*2 + 54)/(2*360 + 360) = 10%. Likewise, if the reject rate of the PA view were higher than that of the LAT view, the image‐based reject rate calculation would be artificially high compared to the true reject rate based on exposure button presses.

### 4.2 Retrofit systems

Digital retrofit systems pose unique challenges to reject analysis. For retrofits, the manufacturer of the x‐ray unit is often different from that of the digital detector, thus generator integration is often partial or non‐existent. Vendor approaches to how detector readout is triggered can be broken down into two broad categories. The first is to tie the readout to the exposure control switch (partial generator integration), such that when the exposure button is pushed, the detector is triggered to readout an image. The second approach uses a self‐sensing detector for which the exposure itself triggers the readout (no generator integration). For either approach, an image will not be produced if an exam tag (a container for the expected image) has not been selected prior to exposure.

Systems that utilize partial generator integration may provide a setting that prohibits exposure if an exam tag is not selected. In the absence of generator integration, exposures cannot be prevented, and it is possible to expose a patient without generating an image. If the system does not generate a DICOM report such as RDSR, there is currently no way to track exposures that do not produce an image, and these remain outside the scope of reject analysis. Otherwise, for all exposures that create images, reject analysis should be implemented within the detector vendor interface.

## 5 IMPLEMENTATION OPTIONS

### 5.1 System‐generated log file(s)

Many current radiography systems are capable of generating and exporting system log files (i.e., text‐based reports) that include information on each acquired and rejected radiograph, though this functionality might come as an add‐on option with an associated cost. While nearly every vendor offers such reports, there is no standardized format and the specifics of the information provided can vary. To the best knowledge of the task group, most file formats can be imported into spreadsheet programs such as Microsoft Excel.

To date, there are two broad categories of how vendors have implemented exposure and reject reports; either as a single file or as separate exposure and reject reports. **We recommend that acquisition and reject information should be provided in a single log file**. The second category, consisting of two separate reports, is not recommended and is described here merely for completeness of existing reports.

*Single file exposure report*. Several vendors produce an exposure report as a single file, with information on each exposure button press included in a single row. Status information such as whether or not the acquisition was rejected is typically included within that row. This type of file format is the most straightforward for reject and exposure analysis. For multi‐acquisitions that produce a series of x‐ray images from a single exposure button press, each individual raw image should be its own row in the exposure report. Multi‐acquisitions must be specified by the “Acquisition Type” field (see Table 1). For example, there may be several rows specified as acquisition type: “Image pasting.”

*Exposure and reject reports as separate files*. Some vendors export exposure and reject information separately. Typically, acquisitions are listed in an exposure report without information on reject status. Both files generally have different formats and provide different information. If one of the files is anonymized, it can be difficult to link the information in the exposure report to that of the reject report. This gives rise to many problems, as illustrated in two use‐case scenarios:

1. If multiple technologists use one radiography system, it might be impossible to count the total number of acquisitions, or the total number of rejects, per technologist, as an accession number would be needed to identify the operator via HIS/RIS data. Alternatively, technologists could use individual logins. However, not all vendors provide such an option, and even if they do, the username is not always included in the exposure report.
2. Another limitation of separate exposure and reject log files arises when the exposure report is to be analyzed for acquisition technique factors. To obtain representative technique information, rejected acquisitions should be excluded from the analysis. For vendor systems that provide insufficient information to link entries in the exposure and reject reports, it is not feasible to exclude rejected acquisitions from that analysis.

#### 5.1.1 Anonymization of log files

**Anonymizing log files is not recommended**. Identifying information should include accession number, or MRN and study date, but not patient name. Identifying information is required when rejected images are reviewed within the context of the entire study, including comparison of images that were rejected with those that were sent to PACS. In addition, if the name of the technologist performing the acquisition is not known to the system (for instance, because a department‐wide login is used), an accession number is required to link the study to information from other clinical data sources, such as the RIS, to identify the performing technologist.

#### 5.1.2 Shortcomings of log files

Even if variation in log files is limited, getting data from each system to a centralized location efficiently can be difficult. Depending on institutional network configuration, it is sometimes possible to send or retrieve log files automatically. However, these implementations often meet resistance from IT security. Most often, files are retrieved from each unit by manually going to the unit and saving the logs to a portable drive, which should also be appropriately encrypted and meet institutional data use policies. **The task group recommends that vendors ensure systems have the capability to automatically export exposure log files to a remote server at regular intervals to facilitate secure data collection and to scale for implementation in large healthcare enterprises**.

### 5.2 DICOM method

It would be advantageous to move reject data from radiography systems to a centralized location using DICOM or DICOMweb, which are methods that are used to transfer other data in radiology. DICOM is ubiquitous in radiology, and methods to route and store DICOM data elements are well‐established in every department that performs radiography.

An advantage of using DICOM would be that reject information from across a healthcare enterprise would be available quickly and without the need to collect the reject/acquisition logs to a central location, which can be time‐consuming if it involves manual data export and import. In addition to metadata from rejected radiographs, the rejected images themselves could easily be stored for review for quality improvement purposes. The availability of reject information (i.e., # of rejects) and actual rejected images from any radiologist's workstation will allow for greater involvement from the radiologist in the QA review process (Figure 3). An Integrating the Healthcare Enterprise (IHE) profile proposal is included on the AAPM webpage for this TG report but not in this article.

This task group supports improving the radiation dose structured report (RDSR) to provide better information on rejected acquisitions. The current RDSR profile, however, is too limited and does not allow for storage of information about anatomy or view, nor the actual images. Many task group members have found the rejected image and various metadata beyond an image's acquisition parameters to be an important part of the quality improvement process. Therefore, we propose an IHE Profile on the AAPM webpage for this report that allows for all of this information to be included as part of a study and to be efficiently transferred to viewing and archiving solutions.

The use of DICOM for reject analysis data transfer has significant hurdles that would need to be overcome. To have the ability to initiate rejects at the acquisition workstation, manufacturers would have to configure their system to send additional flags and to send data to at least one additional destination. Additionally, dose monitoring, workflow engine, or PACS viewer software would need to be modified to make accessing and analyzing the data simple and efficient. Some PACS software vendors already have developed effective solutions for radiation dose monitoring, and these solutions could be adapted to facilitate robust reject analysis. A PACS upgrade to support reject analysis and a change in workflow to only reject images at the PACS could be implemented without any upgrades to the imaging systems. Analysis solutions would not be limited to PACS and dose monitoring companies, however, as the DICOM method would facilitate sending to any potential new analysis software.

## 6 SUMMARY OF RECOMMENDATIONS

- Reject rates should be calculated based on actuations of the x‐ray exposure button, rather than based on individual image counts.
- Coarse‐level reject reasons should be standardized to the structure in Table 3, and detailed‐level reject reasons should be customizable.
- All elements in Tables 1 and 2 should be reported for each acquisition and reject.
- Acquisition and reject information should be provided in a single log file.
- All radiography systems should have the capability to automatically export log files to a remote server at regular intervals.
- The DICOM method outlined in the proposed IHE Profile is preferred, but this is dependent on the implementation of a finalized IHE Profile.

## AUTHOR CONTRIBUTIONS

Kevin Little and Ingrid Reiser, as co‐chairs of AAPM Task Group 305, led the task group meetings that resulted in this report and served as the primary editors of this report. Kevin Little, Ingrid Reiser, Bruce Apgar, Poonam Dalal, Jaydev Dave, Ryan Fisher, Katie Hulme, Mary Ellen Jafari, Emily Marshall, Stephen Meyer, Quentin Moore, Nicole Murphy, Thomas Nishino, Katelyn Nye, Kevin O'Donnell, John Sabol, Adrian Sanchez, William Sensakovic, Lawrence Tarbox, Robert Uzenoff, Alisa Walz‐Flannigan, Charles Willis, and Jie Zhang participated in task group meetings, where the contents of this report were discussed and mutually agreed upon, and contributed to drafting and editing of the report.

## CONFLICT OF INTEREST STATEMENT

(1) The members of TG305 listed below attest that they have no potential Conflicts of Interest related to the subject matter or materials presented in this document. Ingrid Reiser, Ryan Fisher, Katie Hulme, Mary Ellen Jafari, Emily Marshall, Quentin Moore, Nicole Murphy, Thomas Nishino, Adrian Sanchez, William Sensakovic, Lawrence Tarbox, Alisa Walz‐Flannigan, and Jie Zhang. (2) The members of TG305 listed below disclose the following potential Conflict(s) of Interest related to subject matter or materials presented in this document. During the time this work was performed, Kevin Little was an employee of Ohio State University, which has research agreements with Qaelum NV and Siemens Healthineers. Bruce Apgar is an employee of AGFA HealthCare. Jaydev Dave has received research support from Philips Healthcare, Lantheus Medical Imaging Inc., and GE Healthcare. Stephen Meyer is an employee of Canon Medical Components USA. Kevin O'Donnell is an employee of Canon Medical Research USA, a subsidiary of Canon Medical Systems Corporation. Katelyn Nye is an employee of GE Healthcare. Dalal Poonam is an employee of GE Healthcare. During part of the time this work was performed, John Sabol was an employee of GE Healthcare, Robert Uzenoff was an employee of Fujifilm Medical Systems U.S.A., and Charles Willis was a member of the GE Medical Advisory Board for Radiography.

## A1 Definitions

**Acquisition**: The actuation of the x‐ray exposure button. This action can produce one or multiple for‐processing x‐ray images.

**Artifact**: Unwanted elements in the radiograph that do not originate from patient anatomy and may negatively affect the diagnostic quality of the image.^2^

**CR**: Computed radiography. CR produces a digital image when a latent image on a phosphor imaging plate is read out in a CR reader.

**DICOM**: Digital Imaging and Communications in Medicine. International standard to transmit, store, retrieve, print, process, and display medical imaging information (dicomstandard.org). The DICOM file format stores a header and image dataset in a single file.

DICOM header: A list of patient and image attributes (e.g., patient age, pixel size, beam energy) at the beginning of a DICOM file.

**DICOM tag**: A unique identifier for a data element in the DICOM header, composed of a pair of hexadecimal numbers in the format of (xxxx,xxxx).

**DICOMweb**: An extension to the DICOM standard that supports rendering DICOM Objects using JSON and JPEG and performing data transfers using RESTful HTTP messaging.

**DR**: Direct digital radiography. DR produces a digital image quickly without the need to read out a latent image. An indirect or direct x‐ray conversion detector may be used.

**HIS**: Hospital information system. An IT system used for managing patient admission, billing, etc. Some HIS systems also provide an electronic health record (EHR) system.

**IHE**: Integrating the Healthcare Enterprise. An initiative to promote solutions using established standards such as DICOM and HL7. IHE's implementation profiles describe how various standards can be used to solve a clinical need. https://ihe.net

**Image**: See *Radiograph*.

KOS: DICOM Key Object Selection. A DICOM object that serves as a “digital sticky note” that tags one or more images.  DICOM and IHE have profiled usage of these as “Rejection Notes” indicating that images have been rejected and what the reason for rejection is.

**PACS**: Picture archiving and communication system. A computer‐based system used to transfer, store, display, and manage medical images and associated text data.

**PHI**: Protected health information. https://www.hhs.gov/hipaa/for‐professionals/privacy/laws‐regulations/index.html

Presentation state: A DICOM information object definition which describes specific traits regarding the display of images (e.g., labeling, window, level, slice thickness, zoom, field‐of‐view, etc). http://dicom.nema.org/Dicom/2013/output/chtml/part03/sect_A.33.html

**Radiograph or image**: The final product(s) of the acquisition process, that is, the for‐presentation image(s) used for diagnosis. The radiograph (or image) is generally produced from the for‐processing x‐ray images through image processing or synthesis, which might not be presented to the radiologist (for example, as in dual energy radiography).

**Rejected acquisition** *or* **reject** *or* **rejected** **image** *or* **rejected** **radiograph**: Acquisitions of patient anatomy that are discarded by the technologist without being presented for diagnostic interpretation in any form to the radiologist or interpreting physician. An acquisition corresponds to one actuation of the x‐ray exposure button, which can produce one or multiple raw x‐ray images (i.e., for‐processing images). This definition is consistent with AAPM Report 151.^5^ While mammography is out of scope of this report, this definition is consistent with the 1999 Mammography ACR QC manual.^27^ The 2018 Digital Mammography ACR QC manual changed this definition and only uses the term “repeat,” which includes images that were sent for review and those that are discarded without radiologists’ review.^28^ A deleted reprocessed image is only considered a rejected acquisition if no images processed from the acquisition are sent for diagnostic review.

**Reject rate**: Rejected acquisitions divided by total acquisitions, where an acquisition is a single actuation of the exposure button. Reprocessed images are not acquisitions and as such should be excluded from the denominator in this calculation.

**REM**: Radiation Exposure Monitoring IHE Profile

**Repeat** or **repeated** **radiograph** or **re‐take**: Additional radiographs beyond the minimum anatomic views specified in the protocol. All repeats are sent for diagnostic review by a radiologist. One or more of the acquisitions may be considered a supplemental image, where the technologist expects added diagnostic value in the image with an understanding that it will not completely answer the clinical question. While mammography is out of the scope of this report, note that the definition of repeat in the 2018 Mammography ACR QC manual includes both images that are reviewed by radiologists and those that are discarded on the workstation without radiologists’ review.^28^

**Reprocessed image**: An additional image that is produced by changing image processing parameters or cropping the image. A deleted reprocessed image is not considered a rejected acquisition unless no images processed from the acquisition are sent for diagnostic review.

**RIS**: Radiology information system. Computer system used by a radiology department to store, manage, retrieve, and distribute clinical and administrative information.

## A2 Proposed updates to DICOM reject categories.

DICOM currently offers a structured list of reject reasons within context group CID 7011, “Rejected for Quality Reasons” (Table A.1).^22^ Upon inspection of these existing DICOM reject reasons, the task group consensus was that the existing DICOM reject reasons were incomplete, and that based on clinical experience, many DICOM reject reasons are not useful in clinical practice. Therefore an update to the DICOM reject reasons consistent with Table 3, is proposed in Table A.2.

**TABLE A.1:** Existing DICOM reject reasons within context group CID 7011

Image artifact(s)	Mechanical failure
Grid artifact(s)	Electrical failure
Positioning	Software failure
Motion blur	Inappropriate image processing
Underexposed	Other failure
Overexposed	Unknown failure
No image	Double exposure
Detector artifact(s)	Artifact(s) other than grid or detector artifact

Table A.2 proposes DICOM definitions for the broad reject reasons listed in Table 3. Existing DICOM reject definitions are included where they fit the proposed schema. We propose that the DICOM header for a rejected image or rejected image note (KOS) contains two fields, one for the broad reject reason, and one for the customizable detailed reject reason. While we suggest that manufacturers include the detailed categories listed in Table 3 as defaults, the DICOM field for the detailed reason should be a text string to allow for user modifications.

**TABLE A.2:** Proposed updates to DICOM definitions for reject reasons at the coarse level.

Proposed reject categories	DICOM terminology definitions (bold: **proposed**) Code value/code meaning/definition/notes
1. Positioning	111209 Positioning: Inadequate arrangement of the anatomy of interest with respect to the x‐ray field and image detector sensitive area. Examples: 1) positioning is “cutoff” when the projection of anatomy of interest falls outside the sensitive area of the detector; 2) “cone cut,” in which the x‐ray field does not adequately cover the anatomy of interest; 3) detector's sensitive surface is too small to cover the projection of the anatomy of interest; 4) improper angular orientation or “rotation” of anatomy of interest with respect to the x‐ray source, or detector; and 5) projection of other anatomy or clothing over the anatomy of interest in the image.
2. Patient motion	111210 Motion blur: Unacceptable image blur resulting from motion of the anatomy of interest during exposure or the inadequately compensated motion of x‐ray source with respect to the image detector during exposure.
3. Image artifacts	111207 image artifacts: Signals that do not faithfully reproduce actual anatomic structures because of distortion or of addition or deletion of information.
**4. Image contrast or noise**	**Image contrast or noise**: Poor image quality due to factors such as low contrast, unacceptable noise, or pixel value saturation not otherwise due to positioning, motion, or image artifacts. Potential factors include over‐ and underexposure (x‐ray technique factors).
**5. Incorrect selection (protocol, detector, body part, patient)**	**Incorrect selection (protocol, detector, body part, patient)**: The incorrect anatomy, view, or detector was selected on the imaging system, leading to an incorrect acquisition technique, incorrect image processing parameters being applied, or to the patient exposure not being acquired.
**6. Wrong body part or patient imaged**	**Wrong body part or patient imaged**: The body part or patient does not match the ordered exam.
**7. Equipment failure**	**Unspecified equipment failure**: Equipment failed to obtain an adequate image even though it was operated correctly.
**8. Practitioner directed**	**Practitioner directed**: Acquisitions are repeated at the direction of a physician or other practitioner licensed to order x‐ray imaging.
**9. No patient exposure/test**	**No patient exposure/test**: Image was acquired without any patient exposure, such as for quality control or non‐patient research. If acquired for quality control purposes, the image should include DICOM tag (0028,0300), Quality Control Image Attribute, set to YES.
